# Spatial assessment of wolf-dog hybridization in a single breeding period

**DOI:** 10.1038/srep42475

**Published:** 2017-02-14

**Authors:** C. Pacheco, J. V. López-Bao, E. J. García, F. J. Lema, L. Llaneza, V. Palacios, R. Godinho

**Affiliations:** 1 CIBIO/InBio - Centro de Investigação em Biodiversidade e Recursos Genéticos, Universidade do Porto, Campus de Vairão, 4485-661 Vairão, Portugal; 2 Departamento de Biologia, Faculdade de Ciências, Universidade do Porto, Rua do Campo Alegre s ⁄n. 4169-007 Porto, Portugal; 3 Research Unit of Biodiversity, University of Oviedo, 33600, Mieres, Spain; 4 A.RE.NA, Asesores en Recursos Naturales, S.L, 27003 Lugo, Spain; 5 Fuentes Amarelle 6, 15130, Corcubion A Coruña, Spain

## Abstract

Understanding the dynamics of wolf-dog hybridization and delineating evidence-based conservation strategies requires information on the spatial extent of wolf-dog hybridization in real-time, which remains largely unknown. We collected 332 wolf-like scats over ca. 5,000km^2^ in the NW Iberian Peninsula to evaluate wolf-dog hybridization at population level in a single breeding/pup-rearing season. Mitochondrial DNA (MtDNA) and 18 ancestry informative markers were used for species and individual identification, and to detect wolf-dog hybrids. Genetic relatedness was assessed between hybrids and wolves. We identified 130 genotypes, including 67 wolves and 7 hybrids. Three of the hybrids were backcrosses to dog whereas the others were backcrosses to wolf, the latter accounting for a 5.6% rate of introgression into the wolf population. Our results show a previously undocumented scenario of multiple and widespread wolf-dog hybridization events at the population level. However, there is a clear maintenance of wolf genetic identity, as evidenced by the sharp genetic identification of pure individuals, suggesting the resilience of wolf populations to a small amount of hybridization. We consider that real-time population level assessments of hybridization provide a new perspective into the debate on wolf conservation, with particular focus on current management guidelines applied in wolf-dog hybridization events.

Hybridization between wild species and their domestic forms is widely perceived as a biodiversity threat. The outcome of this crossbreeding may lead to the introgression of domestic alleles, shaped by artificial selection, into wild populations, with potential negative conservation consequences, such as genetic homogenization, disruption of local adaptation or, ultimately, extinction^1^,^2^,^3^. However, positive effects of such introgression events have also been recently suggested. For example, the black coat colour present in North American wolves (*Canis lupus*) is the result of a single mutation^4^ that emerged through past interbreeding with dogs, and exhibits a molecular signature of positive selection, apparently confering advantage in forested habitats^5^,^6^.

Hybridization between wolves and dogs is paradigmatic among wild-domestic pairs, attracting a growing attention among managers, researchers and conservationists^3^,^7^,^8^,^9^. Such attention is justified in the current context of wolf population recovery in human-dominated landscapes^10^, where encounters between wolves and either free-ranging, feral or pet dogs are expected to be frequent^11^,^12^,^13^. However, wolf-dog hybrids are not confidently identifiable by morphological features alone. Many countries have thus used molecular tools to study and report wolf-dog hybridization events^14^,^15^,^16^,^17^,^18^. Most of these studies, however, are based on opportunistic invasive sampling that limits the understanding of the spatial and temporal patterns of hybridization at the population level. Opportunistic sampling requires a wide temporal window to achieve a representative sample size, generally encompassing several generations, thereby increasing the time-lag between population survey, interpretation of the dynamics of hybridization and the implementation of interventions.

Non-invasive samples (NIS) overcome some constraints related to opportunistic sampling, but their effective use in wolf-dog hybridization studies has been so far limited by the high genetic similarity between these two entities. In such cases, a large number of molecular markers is needed to achieve accurate results^19^,^20^, but DNA quality and quantity derived from NIS limits the number of markers that can be used^21^. However, using NIS to detect hybridization is now more appealing since recent studies have shown that a selection of a small number of ancestry informative markers (AIMs) is as efficient as a larger dataset of markers not selected based on their discriminatory power^22^,^23^.

The management of wolf-dog hybridization is a controversial issue. First, there is no clear genetic threshold for individual assignment to hybrid classes, which may limit interpretation of what is, in fact, a hybrid. Second, the value and role of contemporary wolf-dog hybrids in ecosystems have not been investigated. Third, in most cases, wild-domestic hybrids are not explicitly addressed in the principal international legal instruments on nature conservation, blurring the boundary for management decisions^7^,^11^,^24^. On top of this, assessments to detect wolf-dog hybridization events at large scales in very short time periods are not available for any wolf population (but see Caniglia *et al*.^25^ for an amalgamation of 9 year surveys), which have so far hampered a real-time estimation of hybridization events at the population level and of hybrid density per wolf generation. Monitoring these parameters properly is not only a way to better understand the dynamics of wolf-dog hybridization or to detect alarming situations, but could also help to define possible thresholds for interventions, as well as to evaluate their effectiveness^23^.

A recent study in NW Galicia, Spain, revealed a dynamic crossbreeding system between Iberian wolves and dogs in a single pack (Barbanza pack^23^, see Fig. 1). Knowing that packs are commonly formed by related individuals^26^, and given the dispersal abilities of wolves, individuals from a hybrid pack could move into adjacent territories possibly mating with other wolves and further introgressing dog genes into the wolf population. In this study, we explore this hypothesis presenting the first real-time spatial assessment of wolf-dog hybridization at a population level. To do so, we rely on a systematic sampling survey for the whole wolf distribution in Costa da Morte (NW Galicia) and on a set of 18 AIMs. Our main goals were to i) determine the spatial pattern of hybridization events in a single year at a population level; ii) identify the genetic composition of admixed individuals, inferring their hybrid class; and iii) assess the relatedness among putative hybrids, and between those and the wolves in the area, eventually identifying the pack of origin for the putative hybrids.

## Results

### Genotyping and individual identification

From the 332 analyzed samples, MtDNA identification was achieved for 301 samples (91%), comprising 172 samples (57%) with the Iberian wolf haplotype W7^27^, 100 samples (33%) matching to 13 different dog haplotypes (see Supplementary Table S3, for more details on the haplotypes), and 29 samples (10%) corresponding to red fox. A total of 272 samples carrying wolf or dog mtDNA were tested for DNA quality from which 91 were excluded. Four replicates of the remaining 181 samples were genotyped for the 18 AIMs. Finally, 167 samples achieved a consensus genotype with <20% missing data (61% of the 272 initial samples), and were considered for further analysis. Estimated allelic dropout across loci was ADO = 0.033 (0 < ADO < 0.103), and false alleles were FA = 0.005 (0 < FA < 0.016; see Supplementary Table S4 for further information). These values are below the average for similar recent non-invasive genetic studies^23^,^25^,^28^,^29^, thereby validating the pre-amplification strategy followed in our work.

We identified 130 individual genotypes, with an average of 4% missing data across loci. This dataset had a probability of identity (PID) = 2.58 × 10^–15^ and a PIDsibs = 2.74 × 10^−6^, with the expected number of sibling individuals sharing an identical genotype by chance of PIDsibs × sample size = 0.000352, meaning that it is very unlikely that identical genotypes do not identify the same individual. Of the 130 individuals, 109 (84%) were only detected once, and the maximum number of recaptures was five for wolves and two for dogs.

### Diversity and differentiation

All loci were polymorphic, showing 2 to 13 alleles per locus for samples from Costa da Morte (CM). Four pairwise combinations of loci showed significant LD in dogs (p < 0.001, corrected for 306 comparisons), whereas none were identified in wolves. Deviations from Hardy-Weinberg expectations were observed in one and four loci in wolves and dogs, respectively (Supplementary Table S5). Iberian wolves exhibited lower genetic diversity than dogs. The mean number of alleles per locus was AN_wolf_ = 3.6 ± 1.8 and AN_dog_ = 6.9 ± 2.8, whereas the mean expected heterozygosity was He_wolf_ = 0.44 ± 0.24 and He_dog_ = 0.69 ± 0.14 (Supplementary Table S5). As expected, the use of AIMs allowed a remarkable genetic differentiation between wolves and dogs (F_ST_ = 0.335; p < 0.001). Loci that contributed more to this observation were DBar1, Cfx30371 and AHT103, which exhibited F_ST_ values > 0.5 (see Supplementary Table S5 for F_ST_ values per locus).

### Hybridization dynamics in Costa da Morte

Bayesian clustering analysis of simulated genotypes revealed high average assignments to the correct parental class, Q*i*_wolf_ = 0.998 (minimum *qi*_wolf_ = 0.964) and Q*i*_dog_ = 0.995 (minimum *qi*_dog_ = 0.894), which allowed us to establish a conservative threshold of *qi* = 0.910 for the assignment to the wolf population and *qi* = 0.850 to the dog population. Based on this threshold, we identified as hybrids 100% of the simulated genotypes for generations F1 (*qi*_F1_ ≤ 0.709) and F2 (*qi*_F2_ ≤ 0.817), 98% and 76% of first generation backcrosses (average *qi*_Bxwolf_ = 0.737, *qi*_BxDog_ = 0.725) and 64% and 31% of second generation backcrosses (average *qi*_2BxWolf_ = 0.866; *qi*_2BxDog_ = 0.841; see Supplementary Fig. S1 for the distribution of *q*-values for the simulated classes).

**Figure 1:**
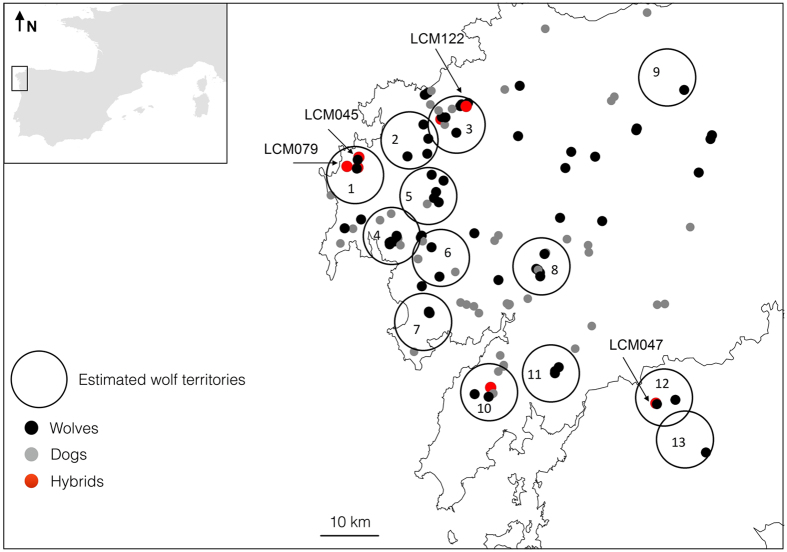
Spatial location of individuals identified as wolf, dog or hybrids in the Bayesian analysis performed in STRUCTURE. Estimated wolf pack territories (100 km^2^; see text for details) are denoted by open circles. Each number represents a pack as follows: 1- Muxia; 2- Vimianzo; 3 - Pasarela; 4 - Buxantes; 5- Baiñas; 6 – Ruña; 7 – Carnota; 8 – Negreira; 9 – Cerceda; 10 – Barbanza; 11 – Lousame; 12 – Xesteiras; 13 – Piorneiras. Inset: Location of the study area within the Iberian Peninsula. Arrows indicate the location of hybrids with *qi* values shifted towards the wolf cluster (LCM045, LCM047, LCM79, LCM122). This figure was produced using ArcGIS (version 10.1 [www.esri.com/software/Arcgis]).

Bayesian clustering analysis performed with CM genotypes showed high posterior probabilities to assign reference wolves and dogs (*Qi*_Wolf_ = 0.996 and *Qi*_Dog_ = 0.995, Table 1). Out of the 130 genotypes considered, we identified 67 wolves (average *qi* = 0.986), and 56 dogs (average *qi* = 0.972) (Table 1, Fig. 1). Seven genotypes were partially assigned to both clusters, with *qi* values shifted towards the wolf cluster for LCM045, LCM047, LCM079, LCM122, and towards the dog cluster for LCM006, LCM068, LCM091 (Table 1). Bayesian credible intervals (BCI, 90%) for these seven hybrids were wide (average range = 0.318) and overlapped with the established thresholds. This is in sharp contrast with non-admixed genotypes, which consistently exhibited narrow BCI values (average range for wolf = 0.044 and dog = 0.106). Considering the percentage of admixed individuals with assignment towards the wolf cluster as a proxy for the rate of dog genome present in the wolf gene pool, we estimate a 5.6% rate of introgression in the sampled wolf population. Hybrid individuals were found in different areas (Fig. 1), occurring within the estimated territory of four different packs (30% of the total number of estimated packs in the study area^30^). However, five of the seven hybrids appeared within contiguous pack territories.

Considering Bayesian ancestry analysis, only hybrid LCM047, with a genomic content shifted towards the wolf cluster, exhibited a high posterior probability of having a dog ancestry going two generations back (Table 2), likely being a first generation backcross to a wolf. The remaining hybrids showed ambiguous posterior probabilities (LCM122, LCM006) or no evidence of having a pure ancestor in the second population (Table 2). Since this model requires powerful data to overcome the prior population assignment and our dataset has a low power to identify hybrids beyond a first generation backcross, the observed posterior probabilities for these individuals might be evidence for multi-generational backcrosses. Interestingly, dog mtDNA haplotypes were observed in hybrids with genomic content shifted towards dog, while hybrids with genomic content shifted towards wolf showed an Iberian wolf mtDNA haplotype (Table 1).

### Relatedness between hybrids and wolves

**Figure 2:**
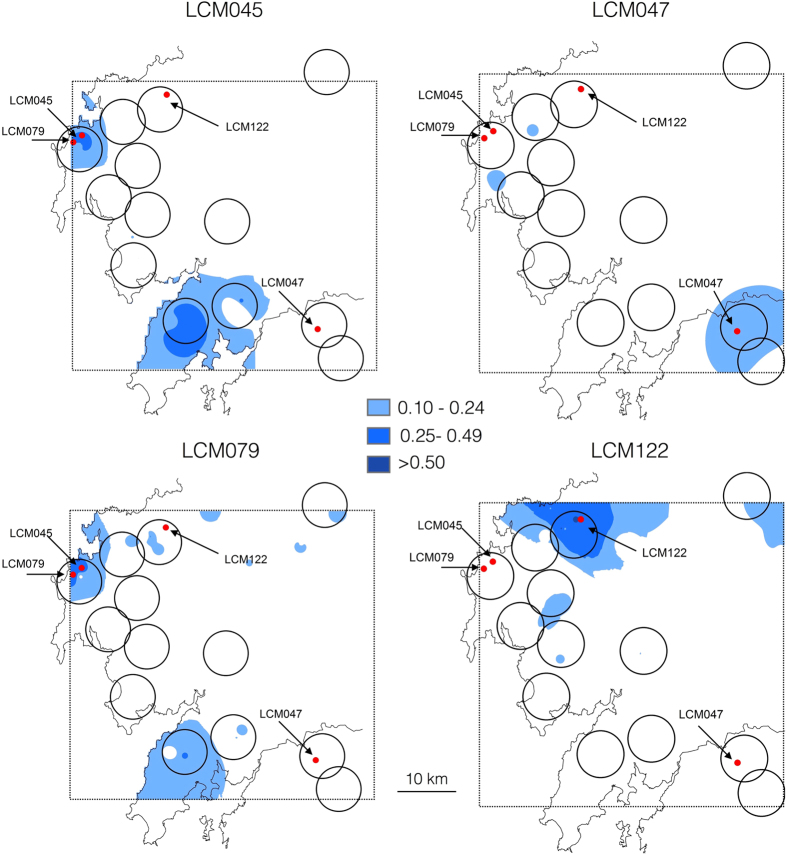
Geographic representation of the interpolated significant relatedness values between each individual identified as backcross to wolf and all wolves sampled in this study. Estimated wolf pack territories (100 km^2^) are denoted by open circles (see Fig. 1 for pack names). This figure was produced using ArcGIS (version 10.1 [www.esri.com/software/Arcgis]).

Pairwise relatedness values between wolves and hybrids with a genomic content shifted towards wolf were higher in pairs sampled in the same area (Fig. 2, Table 3). The mean relatedness of hybrids LCM047, LCM079 and LCM122 to members belonging to the Xesteiras, Muxia and Pasarela packs, respectively, was significantly higher than to the remaining wolf population (Fig. 2, Table 3). Hybrid LCM045 was related to two different packs, Muxia and Barbanza. Interestingly, hybrids LCM045 and LCM079 were the only ones sampled in the same pack territory and presented a pairwise relatedness estimate of *r* = 0.50 (95%CI: 0.1–0.53), consistent with a parent-offspring or full siblings relationship.

## Discussion

In this study, we carried out for the first time a real-time assessment of wolf-dog hybridization at a population scale (covering ca. 5,000 km^2^). We achieved a good compromise among sampling type, relying on NIS, a sampling time restricted to one breeding/pup-rearing period (June-October 2013), and the molecular approach used, which was based on selected AIMs^23^. We overcame the typical constraint associated with the number of markers that can be genotyped when using NIS by selecting the 18 most informative markers differentiating wolves and dogs in NW Galicia from the set of 52 markers available in our laboratory^16^,^23^. We were therefore able to achieve a very high genetic differentiation between species (F_ST_ = 0.335), and a reasonable success rate in identifying hybrid classes F1, F2 and first generation backcrosses. Similar results were obtained previously using a smaller panel of 13 AIMs^23^. However, increasing the number of loci owing to the implemented two-step PCR approach, allowed us to significantly increase the power of individual differentiation (PID and PIDsibs), which is highly valuable for low diversity populations, while decreasing the genotyping errors characteristic of NIS. In fact, the allelic dropout and false allele rates observed in our work were only comparable with studies using invasive sampling (*e.g.* tissue or blood^17^,^31^) or to a recent study using non-invasively collected saliva^32^. A higher number of loci with lower error rates increases the accuracy of results, and thereby helps to overcome the main limitation associated with using low quality DNA to simultaneously detect both parental species and hybrids.

Bayesian analysis of simulated genotypes indicates that wolves and dogs are assigned with posterior probabilities of *qi* > 0.96 and *qi* > 0.90, respectively, despite being generated from reference samples with strict assignments to wolf and dog (*qi* > 0.98). Furthermore, simulated genotypes do not consider additional standing genetic variation to that present in the reference samples. To account for this bias, we established a conservative threshold of qi_wolf_ > 0.91 and qi_dog_ > 0.85, arbitrarily decreasing the threshold values of simulated genotypes by 0.05. This conservative approach ensured detection of pure individuals (wolf or dog), although it could result in underestimating the number of hybrids. Nevertheless, our analysis shows that all F1, F2 and most first generation backcrosses are always correctly assigned as hybrids using our conservative thresholds. Additionally, we observed that the 90% BCI values for hybrids were wider than those found for wolves or dogs. We therefore suggest that the width of the 90% BCIs may be a good proxy for the accurate identification of admixed individuals, as was also suggested by Randi^3^.

We identified a total of 67 wolves and 56 dogs in a survey area covering 5,000 km^2^. This accounts for a minimum number of wolves and dogs of 1.34 and 1.12 individuals/100 km^2^, respectively. However, we believe that the number of dogs might be underestimated because rangers were instructed to avoid dog scats with dog food content while collecting samples. Additionally, dogs were detected in our work throughout the study area in sympatry with wolves (Fig. 1). In fact, free-ranging dogs were often observed during the entire sampling period (authors’ personal observation). This might be interpreted as a measure of human-dominance in the landscape of NW Iberia, and exacerbate the probability of encounters between dogs and wolves, increasing the opportunities for hybridization^3^,^11^,^16^,^33^,^34^.

Bayesian clustering analysis revealed a 5.6% rate of introgression in the wolf population of Costa da Morte. Similar rates of introgression have been previously found in other wolf-dog hybridization studies^16^,^17^,^25^,^35^, although based on invasive sampling and larger temporal scales (but see Caniglia *et al*.^25^). Overall, these results suggest that a low prevalence of hybridization might be a general pattern across wolf populations in the human-dominated landscapes of Europe, regardless of the spatial and temporal scales considered. Despite the occurrence of hybridization, a remarkable proportion of the total genetic variation in CM was partitioned between wolves and dogs (F_ST_ = 0.335, *p* < 0.001). Wolves from CM remained genetically distinct from dogs, a general pattern found in European wolf populations^16^,^17^,^35^, suggesting that effective introgression may be lower than that suggested by the percentage of hybridization cases reported. Accordingly, these observations might be indicative of a remarkable resilience by wolf populations to a small amount of hybridization. If true, hybridization might be buffered by the physiological and behavioral differences between wolves and dogs^3^,^36^,^37^, or more likely by the overall cohesion of wolf social structure. In this case, the inferred hybridization events detected in our study could be a consequence of occasional disruptions of wolf social groups^26^,^38^,^39^,^40^.

Gene flow between wolves and dogs is commonly described as spatially restricted, confined to peripheral wolf ranges or occurring in recently colonized areas^14^,^36^,^41^. However, the NW Iberian Peninsula does not conform with this description^42^. Additionally, admixed individuals were found scattered in the study area and were potentially associated with at least four different packs (Table 3). Furthermore, the lack of relatedness among hybrids, excepting for the two individuals found within the same pack territory, supports more than a single interbreeding cross in CM. Thus, our observation of hybrids does not conform to offspring dispersion after a single hybridization event. Instead, the most plausible scenario is the occurrence of multiple wolf-dog hybridization events over a large spatial area. Our results show, therefore, a new perspective of hybridization dynamics in European wolf populations.

Wolf-dog hybridization is considered as a matter of conservation concern facing fragmented and isolated wolf populations in Europe^9^,^43^,^44^. However, the legal status of wolf-dog hybrids, and the effectiveness of interventions, are still controversial and debated worldwide^7^,^23^,^45^. In addition, the lack of multidisciplinary information on the behavior and role that hybrids play in nature prevents a proper assessment of the risk of hybridization to species conservation^46^. The effective identification of hybrids in the field in close-to real-time is of paramount importance for providing base knowledge on the spatial dimension of hybridization, which is essential for subsequent risk assessments. Our study confirms previous studies about the utility of a reliable, accurate and efficient tool to evaluate wolf-dog hybridization^23^. Current management guidelines state that everything practically possible should be done to remove hybrids from the wild once they have been detected^8^,^43^. However, the effectiveness of removing hybrids remains an issue. For example, Godinho *et al*.^23^ showed that only 44% of the wolf-dog hybrids detected in a hybrid pack were removed after intervention, and in agreement, the present study has confirmed that hybridization was still present in the same area two years later (Barbanza pack, Fig. 1). It could be argued that more efforts in intervention enforcement could have removed all hybrids from this area. However, the spatial extent of the hybridization events reported here, showing multiple hybridization cases occurring over an area of 5000 km^2^, illustrates the limitations in the interventions suggested to manage wolf-dog hybridization.

Additional effort is required to understand other aspects of hybridization beyond genetics, such as behavioral or ecological factors, to gain new insights into the mechanisms buffering the impact of hybridization events or the role of hybrids in nature, both of which carry substantial conservation and management implications. Compiling evidence on the effectiveness of interventions, the evolution of hybridization over time and space, or the ecological role of hybrids, is necessary to promote a new debate about the current management guidelines and practices around wolf-dog hybridization events, including the legal basis for wild hybrids.

## Methods

### Study area and sample collection

Our study area comprises the wolf population of Costa da Morte and surroundings (Galicia, NW Spain), hereafter referred as CM, covering ca. 5,000 km^2^ where 13 packs have been estimated to occur in recent times^30^ (Fig. 1). This is a human-dominated landscape mostly characterized by a patchy and heterogeneous landscape, with sparse human settlements (148 people km^−2^), and wolves feeding frequently on livestock^12^,^42^,^47^. During the 2013 breeding and pup-rearing period^48^ (April-October), 332 wolf-like scats were collected in CM. All scats compatible with wolf (based on shape, size, content, smell)^28^ or dog, excluding scats with dog food, were considered as wolf-like scats. In collaboration with rangers from the Regional Government of Galicia, scats were searched for along transects comprising existing paths, forest trails and firebreaks, with sampling focused on areas distant from human settlements^30^. We sampled a minimum of 10 km per each 10 × 10 km UTM cell, which resulted in a wolf detection probability >0.6 in the study area (based on Bayesian hierarchical-site-occupancy models)^49^, surveying a total of ca. 750 km. Because we sought to detect hybridization events, we relaxed the criteria used previously to identify a wolf-like scats^42^, which increased the number of dog and red fox (*Vulpes vulpes*) scats collected. Scats were georeferenced using a GPS (Fig. 1), and preserved in ethanol 96% at room temperature (20 °C to 25 °C).

### DNA extraction, markers and genotyping

Extraction and manipulation of DNA from fecal samples was confined to dedicated rooms with sterile conditions and positive air pressure. DNA extraction from the 332 scats followed Frantz *et al*.^50^ using the GuSCN/silica procedure^51^. Potential PCR inhibitors were further removed using pre-rinsed Microcon® YM-30 centrifugal Filter Units (Millipore, Billerica, MA). Negative controls were included throughout the process to monitor for potential DNA contamination. Species identification was assessed through mitochondrial DNA control region sequences following Vilà *et al*.^52^. Successful amplifications were sequenced following the BigDye chemistry (Applied Biosystems). Sequencing products were analyzed on an ABI3130xl genetic analyzer (Applied Biosystems) and aligned using SEQSCAPE 2.5 (Applied Biosystems). Sequences from other species other than wolf or dog were excluded from the study.

Individual identification was based on 18 AIMs. These markers were selected from a panel of 52 markers routinely used in our laboratory^16^,^23^ based on F_ST_ values between wolves and dogs (15 markers) and probability of identity (3 markers). The set of markers included 17 microsatellites and a 5 bp deletion at intron 3 of the KIT-ligand gene (KITLG) (Supplementary Table S1). All markers were amplified in two-steps using a pre-amplification protocol^53^. In both steps, AIMs were amplified in four multiplex reactions using the Multiplex PCR Kit (QIAGEN) in a 10 *μ*L final volume. Four replicas of each PCR step were performed. For the first PCR we used approximately 5 ng of DNA and a concentration of 0.2 *μ*M of unlabeled primers. In the second PCR, forward primers were M13-tailed to follow a fluorescent labelling protocol^54^. Thermocycling conditions are given in Supplementary Table S2. DNA quality was assessed by screening four microsatellites (multiplex MP2, Supplementary Table S1) before proceeding to full genotyping. Samples scored with <40% missing data were selected for further amplifications with the remaining loci. PCR products were separated by size on an ABI3130xl genetic analyzer. Alleles were scored against the GeneScan500 LIZ size standard, using genemapper 4.0 (Applied Biosystems) and checked manually twice.

### Data analysis

Two initial replicas of each genotype were used to perform a maximum likelihood estimate of genotyping errors (allelic dropout and false alleles) using pedant 1.0^55^. These estimates were then used to determine the minimum number of replicas needed to minimize genotyping errors using gemini 1.3.0^56^. This number was set to four. Consensus genotypes over four replicas were assembled following Godinho *et al*.^23^: (i) heterozygous genotypes were accepted if the same genotype was observed in two independent PCRs; and (ii) homozygous genotypes were accepted if the genotype was observed in three independent PCRs. Samples with more than 20% missing data were removed from the analysis. Identical genotypes were filtered using gimlet 1.3.3^57^ and discarded. The same software was used to evaluate mean allelic dropout and false allele rates across the 18 loci for the whole dataset, and to estimate the probability of identical genotypes being shared by chance (probability of identity, PID and PIDsibs).

Multilocus genotypes were used to estimate nuclear diversity for wolves and dogs (hybrids were excluded, see below for further details on hybrids identification) based on the number of alleles per locus (AN), and the observed (Ho) and expected (He) heterozygosity for each locus using arlequin 3.5^58^. The same software was used to compute the fixation index F_IS_, estimate departures from Hardy–Weinberg equilibrium (HWE) following Guo and Thompson (1992)^59^ using a test analogous to Fisher’s exact test^58^, and to evaluate significance of association between genotypes at pairs of loci in each population (linkage disequilibrium, LD). Statistical significance was adjusted using sequential Bonferroni corrections. Population differentiation was assessed by Fisher’s exact test, analogues of pairwise mean F_ST_^60^, and an analysis of molecular variance (amova^61^) using arlequin 3.5.

Bayesian clustering analysis implemented in structure 2.3.4^62^,^63^ was used to identify wolves, dogs and possible hybrids by assessing individual membership proportions (*qi*) and their 90% Bayesian Credible Intervals (BCI) to two inferred clusters (K = 2). A panel of 250 Iberian wolves and 230 dogs was used as reference samples to aid the estimation of allelic frequencies. These samples were previously validated using a set of 53 nuclear markers, exhibiting an individual assignment to their putative cluster >98%^23^. The dog reference panel was increased with an additional set of 17 Can de Palleiro breed samples, a autochthonous Galician breed used also as herding dogs. Bayesian clustering analysis was first implemented using the *admixture model* with correlated allele frequencies (Usepopinfo activated for reference samples) in 10 independent runs each with 10^6^ MCMC iterations following a burn-in period of 10^5^ iterations, to guarantee similar posterior probabilities of the data in each run. Assumptions about hybrid ancestry were inferred after the use of the model “*Use population information to test for migrants*”. This model requires prior information on the origin of individuals and assesses the posterior probability of an individual being from i) the *a priori* assigned population, ii) one of the other populations, or iii) having a recent ancestor (parent, grandparent, great-grandparent) from one of the other populations^62^. For instance, a high posterior probability value for having a grandparent in the dog population means that the individual is likely a first generation backcross to wolf. Individuals were assigned *a priori* to the wolf or dog cluster according to *qi* values determined in the admixture model analysis. Admixed individuals were considered to belong to the population with the higher *qi* value. The model was run with MIGRPRIOR = 0.1 and GENBACK = 2. This model is not used to distinguish parental from admixed individuals^62^. Rather, it assumes that the assigned population is usually correct, requiring strong data to contradict this assumption^62^.

The threshold level to differentiate wolves and dogs from hybrids with our set of AIMs was defined based on the power of the admixture analysis to correctly identify simulated individuals with prior known ancestry. We used the reference wolf and dog samples to generate 100 simulated parental genotypes, and these to generate first (F1) and second (F2) generation hybrids, and first (BxW, BxD) and second (BxW2, BxD2) generation backcrosses to wolves and dogs, respectively, using hybridlab 1.0^64^. Simulated genotypes were analyzed in structure 2.3.4, with the *admixture model* and correlated allele frequencies without any prior population information. The result of this analysis allowed estimating the percentage of simulated individuals in each hybrid class that were correctly classified as hybrid.

Calculations of pairwise genetic relatedness (*r*) were performed using the dyadic maximum-likelihood estimator (DyadML^65^) implemented in coancestry 1.0^66^, with 100,000 bootstraps, and using the combined allele frequencies from CM wolves and hybrids plus wolf reference individuals from Galicia. Estimator selection was based on simulation results (see Appendix S1). Estimations were performed for all wolves and hybrids with a genomic content shifted towards wolf, from CM samples. First, pairwise relatedness was estimated for all pairs of individuals. Second, we compared pairwise relatedness within hybrid individuals. Finally, we compared relatedness between each hybrid and potential wolf pack members. To do this, we linked wolf samples with estimated pack territories. Pack territories were estimated by creating a conservative 100 km^2^ buffer area centered on the rendezvous sites of known packs in the area^67^ (Fig. 1). The selection of the size of the buffer area was based on the wolf home range size of adult wolves in the study area^30^. We clustered all wolf samples within each estimated pack range. We then compared the mean relatedness *r* of each hybrid-pack dyad using the test for group differences implemented in coancestry 1.0, with 100,000 bootstraps. A significant result would suggest that a given hybrid was more closely related to that pack compared to the rest of the population, indicating a potential natal pack. The spatial distribution of relatedness values between each hybrid-wolf pair was also explored through interpolations using the inverse distance-weighted interpolation algorithm implemented in ArcGIS 10.0 (ESRI Inc., Redlands, CA, USA) to produce a continuous surface of relatedness variation over the study area.

## Additional Information

**How to cite this article**: Pacheco, C. *et al*. Spatial assessment of wolf-dog hybridization in a single breeding period. *Sci. Rep.* **7**, 42475; doi: 10.1038/srep42475 (2017).

**Publisher's note:** Springer Nature remains neutral with regard to jurisdictional claims in published maps and institutional affiliations.

## Supplementary Material

Supplementary Information

## Figures and Tables

**Table 1:** Average individual assignment (*Qi*) and *qi* range for simulated, reference and parental CM genotypes; Individual assignment (*qi*) and 90% Bayesian Credible Intervals (BCI) inferred for the seven hybrids.

Sample			Wolf	
			*Qi*	*qi range*
Simulated wolves			0.998	0.964–0.999
Simulated dogs			0.005	0.001–0.106
Reference wolves			0.996	0.968–0.999
Reference dogs			0.005	0.001–0.070
CM Wolves			0.986	0.916–0.999
CM Dogs			0.028	0.002–0.138
			**Wolf**	
**Sample**	**Sex**	**MtDNA**	***qi***	**BCI 90%**
LCM006	F	Dog	0.292	(0.083, 0.508)
LCM045	F	Wolf	0.880	(0.750, 0.973)
LCM047	M	Wolf	0.744	(0.585, 0.878)
LCM068	M	Dog	0.167	(0.000, 0.442)
LCM079	F	Wolf	0.830	(0.661, 0.956)
LCM091	M	Dog	0.181	(0.038, 0.368)
LCM122	M	Wolf	0.890	(0.761, 0.980)

Sex and information on mitochondrial genomic assignment is presented for the hybrids. Individual assignment (*qi*) was calculated in STRUCTURE considering two clusters (K = 2).

**Table 2:** Inferred ancestry of the seven hybrids observed in this study using the *population information model* in STRUCTURE.

Hybrid	prior pop	*q* prior pop	*q* other pop			Probable Ancestry
			Dog	Dog parent	Dog grandparent	
LCM045	Wolf	0.988	0	0	0.012	>1^st^ Bx
LCM047	Wolf	0.002	0	0	**0.998**	1^st^ Bx
LCM079	Wolf	0.909	0	0	0.091	>1^st^ Bx
LCM122	Wolf	0.604	0	0	**0.396**	>1^st^ Bx
			**Wolf**	**Wolf parent**	**Wolf grandparent**	
LCM006	Dog	0.631	0	0	**0.369**	>1^st^ Bx
LCM068	Dog	0.865	0	0	0.135	>1^st^ Bx
LCM091	Dog	0.962	0	0	0.038	>1^st^ Bx

We show estimates of posterior probabilities (*q)* for each individual to have ancestry in its *a priori* assigned population (*q* prior pop), or in the other population in the present generation (dog or wolf), in the first (parent) past generation or the second (grandparent) past generation.

**Table 3:** Mean relatedness of significant hybrid-pack dyad for each individual identified as backcross to wolf, calculated using the test for group differences implemented in COANCESTRY 1.0.

Hybrid	Sampling pack	Related pack	Mean *r* with related pack	Mean *r* with other wolves	*p* value
LCM122	Pasarela	Pasarela	0.292 ± 0.043, n = 11	0.037 ± 0.004, n = 59	*p* < 0.001
LCM079	Muxia	Muxia	0.293 ± 0.031, n = 3	0.040 ± 0.004, n = 64	*p* < 0.001
LCM047	Xesteiras	Xesteiras	0.177 ± 0.001, n = 3	0.013 ± 0.001, n = 64	*p* < 0.001
LCM045	Muxia	Muxia	0.275 ± 0.030, n = 3	0.028 ± 0.006, n = 64	*p* < 0.001
		Barbanza	0.346 ± 0.020, n = 3	0.030 ± 0.006, n = 64	*p* < 0.001

The pack territory in which the individual was sampled is identified as “Sampling pack”, and the pack most closely related with each hybrid is identified as “Related pack”.
