# Supplementary material for: Spatial assessment of wolf-dog hybridization in a single breeding period
Source: Sci Rep. 2017 Feb 14;7:42475. doi: 10.1038/srep42475 (PMC5307949; doi:10.1038/srep42475)
Supplement: Supplementary Information [file srep42475-s1.doc]

**Spatial assessment of wolf-dog hybridization in a single breeding period**

Pacheco C, López-Bao JV, Garcia E, Lema FJ, Llaneza L, Palacios V, Godinho R

**Table S1.** Nuclear loci analysed in this study, microsatellite repeat type, allele range, multiplex protocol used for PCR amplification and references.

| **Microsatellite** | **Repeat** | **Allele Range** | **Multiplex** | **Reference** |
| --- | --- | --- | --- | --- |
| AHT103 | Di | 71-89 | Mp3 | Holmes *et al.* 1995 |
| AHT111 | Di | 72-92 | Mp3 | Holmes *et al.* 1993 |
| AHT121 | Di | 74-118 | Mp1 | Holmes *et al.* 1995 |
| AHTh171 | Di | 216-240 | Mp4 | Breen *et al.* 2001 |
| AHTk211 | Di | 82-98 | Mp1 | Lingaas et al. 1997 |
| C09.173 | Di | 100-118 | Mp4 | Ostrander *et al.* 1993 |
| C09.474 | Di | 111-133 | Mp2 | Ostrander *et al.* 1995 |
| C20.253 | Di | 95-125 | Mp3 | Ostrander *et al.* 1993 |
| C22.279 | Di | 108-132 | Mp4 | Ostrander *et al.* 1993 |
| C27.442 | Di | 158-172 | Mp3 | Ostrander *et al.* 1995 |
| CPH2 | Di | 87-113 | Mp2 | Fredholm & Winterø 1995 |
| CPH9 | Di | 133-163 | Mp2 | Fredholm & Winterø 1995 |
| Cfx30371 | Tetra | 125-161 | Mp2 | Godinho *et al.* 2015 |
| Dbar1 | Di | 183-274 | Mp3 | Kerns *et al.* 2004 |
| FHC2010 | Tetra | 216-240 | Mp4 | Francisco *et al.* 1996 |
| INU030 | Di | 136-156 | Mp1 | Finnzymes, Inc |
| REN162C04 | Di | 189-215 | Mp1 | Guyon *et al.* 2003 |
| **Indel** | **Size** | **Sequence** | **Multiplex** | **Reference** |
| KITLG.indel | 5bp | CAGCA | Mp2 | Godinho *et al*. 2015 |

**Table S2.** Nuclear markers, multiplex protocol, dye and PCR conditions for the amplification of the 18 loci.

|  | **Multiplex** | **Dye** | **Annealing temperature/time** | **Extension time** | **N cycles** |
| --- | --- | --- | --- | --- | --- |
| **AHT121** | Mp1 | Pet | 58ºC/ 60 sec | 30 sec | 40 |
| **AHTk211** |  | Fam |  |  |  |
| **INU030** |  | Ned |  |  |  |
| **REN162C04** |  | Pet |  |  |  |
|  |  |  |  |  |  |
| **C09.474** | Mp2 | Pet | 58ºC/ 60 sec | 30 sec | 40 |
| **CPH2** |  | Ned |  |  |  |
| **CPH9** |  | Ned |  |  |  |
| **Cfx30371** |  | Vic |  |  |  |
| **KITLG.indel** |  | Vic |  |  |  |
|  |  |  |  |  |  |
| **AHT103** | Mp3 | Ned | 58ºC/ 60 sec | 30 sec | 40 |
| **AHT111** |  | Vic |  |  |  |
| **C20.253** |  | Pet |  |  |  |
| **C27.442** |  | Pet |  |  |  |
| **Dbar1** |  | Vic |  |  |  |
|  |  |  |  |  |  |
| **FHC2010** | Mp4 | Fam | 58ºC/ 60 sec | 30 sec | 40 |
| **AHTh171** |  | Pet |  |  |  |
| **C09.173** |  | Ned |  |  |  |
| **C22.279** |  | Fam |  |  |  |

**Table S3**. Dog mtDNA haplotypes observed in this study and respective references. “N samples” refers to the number of samples that exhibited each haplotype, for a total of 100 samples. Please note that the number of samples does not represent the number of individuals (only samples with sufficient DNA quality were genotyped for microsatellites).

| **N Samples** | **Haplotype** | **Reference** |
| --- | --- | --- |
| 24 | A17 | Webb and Allard, 2009 |
| 16 | B1 | Webb and Allard, 2009 |
| 16 | A11 | Webb and Allard, 2009 |
| 15 | A18 | Webb and Allard, 2009 |
| 14 | C2 | Webb and Allard, 2009 |
| 5 | H15 | Pires *et al*. 2006 |
| 2 | C3 | Webb and Allard, 2009 |
| 2 | A22 | Webb and Allard, 2009 |
| 2 | A71 | Webb and Allard, 2009 |
| 1 | H31 | Pires *et al*. 2006 |
| 1 | Be44 | Verscheure *et al.* 2014 |
| 1 | Be45 | Verscheure *et al.* 2014 |
| 1 | A27 | Webb and Allard, 2009 |

**Table S4.** Probability of Identity among all individuals (PID) and among siblings (PIDsibs) for each locus used in this study, and for all loci combined.

| **Locus** | **Dropout** | **False allele** | **PID** | **PIDsibs** |
| --- | --- | --- | --- | --- |
| AHT103 | 0.071 | 0.000 | 0.254 | 0.552 |
| AHT111 | 0.005 | 0.000 | 0.092 | 0.393 |
| AHT121 | 0.009 | 0.000 | 0.041 | 0.341 |
| AHTh171 | 0.103 | 0.008 | 0.108 | 0.430 |
| AHTk211 | 0.000 | 0.000 | 0.225 | 0.532 |
| C09.173 | 0.006 | 0.012 | 0.077 | 0.379 |
| C09.474 | 0.000 | 0.000 | 0.146 | 0.458 |
| C20.253 | 0.028 | 0.000 | 0.154 | 0.454 |
| C22.279 | 0.008 | 0.000 | 0.053 | 0.353 |
| C27.442 | 0.031 | 0.010 | 0.082 | 0.384 |
| Cfx30371 | 0.000 | 0.000 | 5.788 | 2.740 |
| CPH2 | 0.000 | 0.000 | 0.145 | 0.456 |
| CPH9 | 0.039 | 0.016 | 0.123 | 0.427 |
| Dbar1 | 0.100 | 0.009 | 0.145 | 0.464 |
| FHC2010 | 0.068 | 0.023 | 0.163 | 0.457 |
| INU030 | 0.062 | 0.008 | 0.074 | 0.379 |
| KITLG.indel | 0.000 | 0.000 | 0.677 | 0.828 |
| REN162C04 | 0.064 | 0.000 | 0.120 | 0.427 |
|  |  |  |  |  |
| **All loci** | 0.033 | 0.005 | 2.588E-15 | 2.743E-06 |

**Table S5.** Number of gene copies analysed, alleles per locus (Na), observed (Ho), expected (He) heterozygosity and Fis for wolves and dogs; Total number of alleles (AN total) and Fst are presented for the whole dataset. All values are also presented for all loci combined. Values were estimated using only the Costa da Morte samples. *Loci with deviations from Hardy-Weinberg equilibrium; #Loci used for increasing the power of individual identification. s.d. = standard deviation.

| **Locus** | **Dog** | | | | |  | **Iberian wolf** | | | | |  | **AN total** | **Fst** |
| --- | --- | --- | --- | --- | --- | --- | --- | --- | --- | --- | --- | --- | --- | --- |
|  | N gene copies | AN | Ho | He | FIS |  | N gene copies | AN | Ho | He | FIS |  |  |  |
| C09.474 | 110 | 6 | 0.564 | 0.712 | 0.210 |  | 134 | 3 | 0.358 | 0.333 | -0.076 |  | 7 | 0.396 |
| CPH2 | 110 | 8 | 0.655 | 0.694 | 0.057 |  | 134 | 3 | 0.284 | 0.363 | 0.220 |  | 8 | 0.394 |
| CPH9 | 110 | 7 | 0.655 | 0.712 | 0.081 |  | 134 | 4 | 0.478 | 0.530 | 0.100 |  | 9 | 0.242 |
| Cfx30371 | 70 | 2 | 0.533 | 0.474 | -0.037 |  | 134 | 1 | n.a. | n.a. | n.a. |  | 2 | 0.612 |
| KITLG.indel | 110 | 2 | 0.236 | 0.354 | 0.335 |  | 134 | 2 | 0.015 | 0.015 | 0.000 |  | 2 | 0.216 |
| AHT103 | 110 | 5 | 0.564 | 0.741 | 0.242 |  | 134 | 2 | 0.015 | 0.015 | 0.000 |  | 5 | 0.524 |
| AHT111 | 110 | 8 | 0.491 | 0.668 | 0.267 |  | 134 | 4 | 0.507 | 0.577 | 0.121 |  | 8 | 0.321 |
| C20.253^#^ | 110 | 6 | 0.291 | 0.392 | 0.260 |  | 134 | 4 | 0.657 | 0.640 | -0.026 |  | 6 | 0.348 |
| C27.442 | 110 | 7 | 0.600 | 0.723 | 0.172 |  | 134 | 5 | 0.522 | 0.552 | 0.054 |  | 8 | 0.322 |
| Dbar1 | 110 | 10 | 0.455 | 0.780* | 0.420 |  | 134 | 1 | n.a. | n.a. | n.a. |  | 10 | 0.621 |
| AHT121^#^ | 108 | 12 | 0.685 | 0.806 | 0.151 |  | 134 | 8 | 0.612 | 0.760 | 0.196 |  | 13 | 0.139 |
| AHTh171 | 92 | 13 | 0.587 | 0.878* | 0.334 |  | 118 | 5 | 0.339 | 0.367 | 0.076 |  | 13 | 0.268 |
| AHTk211 | 106 | 5 | 0.396 | 0.782* | 0.496 |  | 134 | 2 | 0.045 | 0.072 | 0.383 |  | 5 | 0.452 |
| INU030^#^ | 108 | 7 | 0.648 | 0.764 | 0.153 |  | 134 | 5 | 0.522 | 0.587 | 0.110 |  | 8 | 0.254 |
| REN162C04 | 90 | 8 | 0.378 | 0.755* | 0.502 |  | 122 | 5 | 0.377 | 0.366 | -0.030 |  | 9 | 0.401 |
| C09.173 | 98 | 6 | 0.612 | 0.793 | 0.230 |  | 128 | 3 | 0.641 | 0.636 | -0.007 |  | 6 | 0.181 |
| C22.279 | 110 | 7 | 0.673 | 0.789 | 0.148 |  | 134 | 5 | 0.821 | 0.718 | -0.144 |  | 7 | 0.167 |
| FHC2010 | 96 | 6 | 0.563 | 0.686 | 0.182 |  | 132 | 3 | 0.348 | 0.519* | 0.331 |  | 6 | 0.204 |
|  |  |  |  |  |  |  |  |  |  |  |  |  |  |  |
| **Overall loci** | 104 | 6.9 | 0.532 | 0.695 | 0.215 |  | 132 | 3.6 | 0.409 | 0.441 | 0.059 |  | 7 | 0.335 |
| **s.d.** | 11 | 2.8 | 0.133 | 0.143 | n.a. |  | 5 | 1.8 | 0.236 | 0.238 | n.a. |  | 3 | n.a. |

**Figure S1.** Distribution of *qi* values on simulated genotypes for each hybrid class (F1, F2, 1^st^ generation backcross to wolf (1stBxW), 1^st^ generation backcross to dog (1stBxD), 2^nd^ generation backcross to wolf (2stBxW), 2^nd^ generation backcross to dog (2stBxD)). Threshold values to wolf and dog cluster assignments are denoted by the upper and lower dashed-lines, respectively.

**
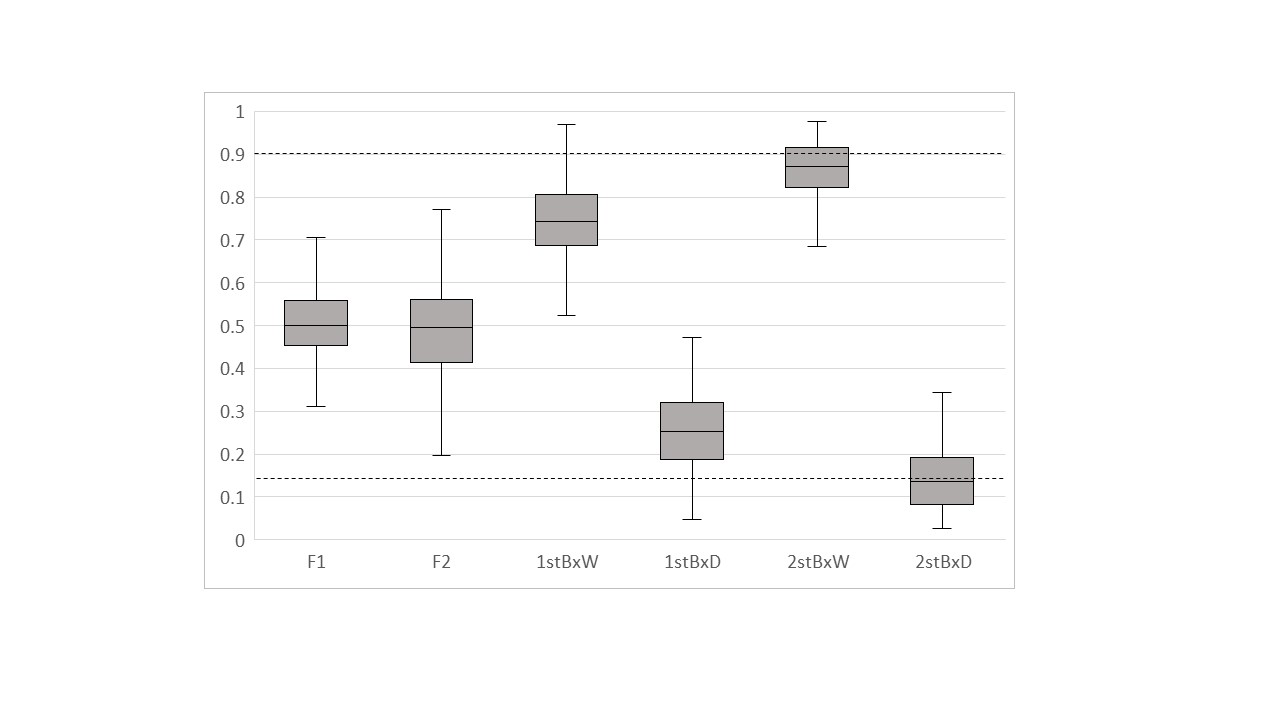
**

**Appendix S1.** Details on relatedness analysis

**Marker-based relatedness simulations.**

Simulations using the software coancestry 1.0 (Wang 2011) were used to assess the performance of different marked-based relatedness estimators. Genotypes were simulated relying on the empirical allele frequencies, missing data and error rates across the 18 microsatellite markers from Costa da Morte. The simulated dataset consisted of 100 dyads spread equally across five categories of relatedness: parent-offspring (r_xy_ = 0.5), full sibling (r_xy_ = 0.5), half siblings/ grandparent–grandchild (r_xy_ = 0.25), first cousins (r_xy_ = 0.125), second cousins (r_xy_ = 0.030), and unrelated (r_xy_ = 0). The seven estimators implemented in coancestry 1.0 were used to assess the relatedness coefficient of the simulated dataset:

- Two likelihood estimators: the triadic likelihood estimator (TrioML; Wang 2007) and the dyadic likelihood estimator (DyadML; Milligan 2003), which estimate the probability of individuals being included in a particular relationship category given the marker data available;
- Five moment estimators: Queller & Goodnight (1989), Lynch (1988) and Li *et al*. (1993), Ritland (1996), Lynch & Ritland (1999) and Wang (2002), which estimate the relatedness based on the probability of identity by descent (IBD).

Relatedness of the simulated genotypes was assessed with the allele frequencies from the simulated dataset, using 200 reference individuals, 10,000 bootstrap replicates, and taking into account genotyping error rates.

| **True Relationship** | **Actual R_xy_** | **Mean R_xy_ Queller&**  **Goodnight** | **95% CI** | **Mean R_xy_ Li** | **95% CI** | **Mean R_xy_ Ritland** | **95% CI** | **Mean R_xy_ Lynch&**  **Ritland** | **95% CI** | **Mean R_xy_ Wang** | **95% CI** |
| --- | --- | --- | --- | --- | --- | --- | --- | --- | --- | --- | --- |
| **Parent-offspring** | 0.500 | 0.436 | (0.249;0.624) | 0.438 | (0.249;0.633) | 0.342 | (0.098;0.668) | 0.363 | (0.157;0.638) | 0.438 | (0.264;0.615) |
| **Full sibling** | 0.500 | 0.436 | (0.171;0.657) | 0.438 | (0.18;0.666) | 0.343 | (0.076;0.699) | 0.364 | (0.122;0.656) | 0.438 | (0.185;0.661) |
| **Half Siblings** | 0.250 | 0.200 | (-0.059;0.44) | 0.209 | (-0.061;0.458) | 0.150 | (-0.056;0.395) | 0.183 | (-0.011;0.425) | 0.214 | (-0.047;0.454) |
| **First cousin** | 0.125 | 0.093 | (-0.166;0.346) | 0.093 | (-0.179;0.352) | 0.078 | (-0.1;0.289) | 0.090 | (-0.086;0.305) | 0.097 | (-0.165;0.348) |
| **Second cousin** | 0.030 | 0.039 | (-0.209;0.293) | 0.039 | (-0.237;0.307) | 0.027 | (-0.134;0.209) | 0.031 | (-0.115;0.21) | 0.028 | (-0.231;0.289) |
| **Unrelated** | 0 | 0.019 | (-0.223;0.266) | 0.031 | (-0.246;0.296) | -0.002 | (-0.14;0.141) | 0.010 | (-0.119;0.164) | 0.042 | (-0.226;0.3) |

**Table S6**. Comparison between the true relatedness coefficient of simulated dyads (Actual Rxy) with the average relatedness coefficient and 95% CI values of the 100 simulated genotypes for each relationship category assessed with the seven estimators implemented in COANCESTRY 1.0. A) likelihood estimators, B) moment estimators.

| **True Relationship** | **Actual R_xy_** | **Mean R_xy_ TrioML** | **95% CI** | **Mean R_xy_ DyadML** | **95% CI** |
| --- | --- | --- | --- | --- | --- |
| **Parent-offspring** | 0.500 | 0.461 | (0.341; 0.655) | 0.499 | (0.315; 0.655) |
| **Full sibling** | 0.500 | 0.462 | (0.247; 0.716) | 0.499 | (0.214; 0.727) |
| **Half Siblings** | 0.250 | 0.217 | (0.067; 0.514) | 0.255 | (0.041; 0.508) |
| **First cousin** | 0.125 | 0.138 | (0.014; 0.397) | 0.140 | (0.004; 0.4) |
| **Second cousin** | 0.030 | 0.065 | (0.004; 0.343) | 0.085 | (0.001; 0.336) |
| **Unrelated** | 0 | 0.045 | (0.004; 0.322) | 0.062 | (0.001; 0.319) |

**A.**

**B.**

When comparing the mean relatedness estimations with the real relatedness coefficient, the likelihood estimators showed better estimations of relatedness than moment estimators, especially in the higher relationship categories (Table S6). Accordingly, Pearson’s correlation coefficients reported by coancestry 1.0 were higher for the two likelihood estimators of 0.87 and 0.88, for the TrioML and DyadML estimators, respectively.

To further explore the different behaviour of the two likelihood estimators, we determined the success rate in correctly identifying the true relatedness of the simulated dyad.

**Table S7**. Percentage of correct identification of the two likelihood estimator implemented in coancestry.

|  | **Actual R_xy_** | **TrioML** | **DyadML** |
| --- | --- | --- | --- |
| **Parent-offspring** | 0.500 | 68% | 86% |
| **Full sibling** | 0.500 | 53% | 63% |
| **Half Siblings** | 0.250 | 42% | 53% |
| **Unrelated** | 0 | 51% | 57% |

Results in Table S7 indicate that the DyadML estimator had a higher percentage in the correct identification of the dyad relatedness coefficient than the TrioML estimator. Therefore, based on these results it is possible to conclude that the DyadML estimator will provide the most accurate estimations of Rxy when applied to an empirical dataset.

However, we note that lower relationship categories (first cousin, second cousin and unrelated) can be slightly overestimated (Table S6), and thus we suggest caution in the interpretation of these categories. Nonetheless, coancestry 1.0 estimations of the parentage exclusion probability when one parent or no parent of the offspring is known, is 0.99 and 1, respectively, for all loci, indicating that with our set of AIMs the probability of identifying an unrelated individual as a parent is negligible.

Additionally, it is important to mention that the observed confidence intervals for the relatedness estimations of the simulated dyads are wide and overlapping in general. This is particularly evident in lower relationship categories, with the parent-offspring dyads presenting the narrowest confidence intervals. Nevertheless, these results are consistent with expectations when allele frequencies present a Dirichlet distribution, as our data show (Milligan 2003).

**References**

Breen, M. *et al.* Chromosome-specific single-locus FISH probes allow anchorage of an 1800-marker integrated radiation-hybrid/linkage map of the domestic dog genome to all chromosomes. *Genome Research* **11**, 1784–1795 (2001).

Francisco, L. V., Langsten, A. A., Mellersh, C. S., Neal, C. L. & Ostrander, E. A. A class of highly polymorphic tetranucleotide repeats for canine genetic mapping. *Mammalian Genome* **7**, 359–362 (1996).

Fredholm, M. & Winterø, A. K. Variation of short tandem repeats within and between species belonging to the Canidae family. *Mammalian Genome* **6**, 11–18 (1995).

Godinho, R. *et al.* Real‐time assessment of hybridization between wolves and dogs: combining noninvasive samples with ancestry informative markers. *Molecular Ecology Resources* **15**, 317–328 (2015).

Guyon, R. *et al.* A 1-Mb resolution radiation hybrid map of the canine genome. *Proceedings of the National Academy of Sciences* **100**, 5296–5301 (2003).

Holmes, N. G. *et al.* Eighteen canine microsatellites. *Animal Genetics* **26**, 132–133 (1995).

Holmes, N. G. *et al.* Isolation and characterization of microsatellites from the canine genome. *Animal genetics* **24**, 289–292 (1993).

Kerns, J. A. *et al.* Characterization of the dog Agouti gene and a nonagoutimutation in German Shepherd Dogs. *Mammalian Genome* **15**, 798–808 (2004).

Li, C. C., Weeks, D.E. & Chakravarti, A. Similarity of DNA Fingerprints Due to Chance and Relatedness. *Human Heredity* **43**, 45-52 (1993).

Lingaas, F. *et al.* Towards construction of a canine linkage map: establishment of 16 linkage groups. *Mammalian Genome* **8**, 218-221 (1997).

Lynch, M. Estimation of relatedness by DNA fingerprinting. *Molecular Biology and Evolution* **5**, 584-599 (1988).

Lynch, M. & Ritland, K. Estimation of pairwise relatedness with molecular markers. *Genetics* **152**, 1753–1766 (1999).

Milligan, B. G. Maximum-likelihood estimation of relatedness. *Genetics* **163**, 1153–1167 (2003).

Ostrander, E. A., Mapa, F. A., Yee, M. & Rine, J. One hundred and one new simple sequence repeat-based markers for the canine genome. *Mammalian Genome* **6**, 192–195 (1995).

Ostrander, E.A., Sprague Jr, G.F. & Rine, J. Identification and Characterization of Dinucleotide Repeat (CA)^n^ Markers for Genetic Mapping in Dog. *Genomics* **16**, 207–213 (1993).

Pires, A. E., *et al.* Mitochondrial DNA sequence variation in Portuguese native dog breeds: diversity and phylogenetic affinities. *Journal of Heredity*  **97**, 318-330 (2006).

Queller, D. C. & Goodnight, K. F. Estimating relatedness using genetic markers. *Evolution* **43**, 258–275 (1989).

Verscheure, S., Backeljau, T. & Desmyter, S. Dog mitochondrial genome sequencing to enhance dog mtDNA discrimination power in forensic casework. *Forensic Science International: Genetics* **12**, 60-68 (2014).

Wang, J. Triadic IBD coefficients and applications to estimating pairwise relatedness. *Genetical research* **89**, 135–153 (2007).

Wang, J. An estimator for pairwise relatedness using molecular markers. *Genetics* **160**, 1203–1215 (2002).

Wang, J. coancestry: a program for simulating, estimating and analysing relatedness and inbreeding coefficients. *Molecular Ecology Resources* **11**, 141–145 (2011).

Webb, K. M., Allard M. W. Mitochondrial genome DNA analysis of the domestic dog: identifying informative SNPs outside of the control region. *Journal of forensic sciences* **54**, 275-288 (2009).
